# A novel reaction time assessment in virtual reality: Advantages over computerized tests

**DOI:** 10.3758/s13428-025-02752-w

**Published:** 2025-07-16

**Authors:** Topaz Loushy Kay, Ella Been, Chaim G. Pick

**Affiliations:** 1 Department of Anatomy and Anthropology, School of Medicine, Gray Faculty of Medical and Health Sciences, Tel-Aviv University, Tel Aviv, Israel; 2 Sylvan Adams Sports Science Institute, Tel Aviv University, Tel Aviv, Israel; 3 Department of Sports Therapy, Faculty of Health Professions, Ono Academic College, Kiryat Ono, Israel; 4 Sagol School of Neuroscience, Tel Aviv University, Tel Aviv, Israel; 5 Dr. Miriam and Sheldon G. Adelson Chair and Center for the Biology of Addictive Diseases, Tel Aviv University, Tel Aviv, Israel

**Keywords:** Choice reaction time, Kinematics, Movement time, Movement velocity, Spatial uncertainty, Moving stimuli

## Abstract

Reaction time (RT) is a fundamental cognitive function impacting daily life and sports activities. Despite the increasing use of virtual reality (VR) for cognitive assessments, its efficacy for RT assessments remains uncertain. We aimed to develop and validate a novel RT test in VR, and to explore the potential contributions of VR technology to RT assessment. Forty-eight participants completed a computerized RT test (COM-RT) and a novel RT test in VR (VR-RT). The latter replicated the COM-RT test, assessing simple and choice RTs. Additionally, it introduced more complex conditions: reaching to touch static stimuli in known or unknown locations or moving stimuli. Correlations and differences between the tests and tasks were examined. Moderate-to-strong linear correlations were found between the tests for the simple (*r* ≥ 0.642) and choice (*r* ≥ 0.736) tasks, and the difference between them (*r* ≥ 0.677) (*p* < 0.001). However, RTs were significantly longer in the VR-RT test compared with the COM-RT test (*p* < 0.001). Significant differences were found among the RT-VR tasks (*p* < 0.001): reaching to touch stimuli involved longer RTs compared with pressing a button, and RT was even longer when stimuli appeared in unexpected locations. Moving stimuli were associated with shorter RT. Additionally, movement velocity was significantly higher when reaching for dynamic versus static stimuli in known or unknown locations (*p* < 0.001). VR is valid for RT measurement, yet its outcomes should be interpreted within its framework rather than in comparison to computer assessments. Furthermore, VR offers additional possibilities, such as using lifelike stimuli and measuring kinematics.

**Supplementary Information:**

The online version contains supplementary material available at 10.3758/s13428-025-02752-w.

## Introduction

Despite the widespread adoption of new technologies in various aspects of life, cognitive testing has largely remained outdated, primarily relying on traditional assessments (Asensio & Duñabeitia, 2023; Neguţ et al., 2016b). Currently, there is a lack of widely accepted standardized cognitive tests that leverage new technologies with established normative data (Neguţ et al., 2016b).

Reaction time (RT) is a fundamental cognitive function that significantly impacts daily activities and performance across various professional and sports domains (Janicijevic & Garcia-Ramos, 2022). RT can be divided into two components: decision time, which is the time interval required to decide on a motor response, and movement time (MT), which is the time interval needed to execute the decided movement (Schneider & McGrew, 2012).

Two types of RT are extensively studied in the research literature: simple RT (SRT) and choice RT (CRT). SRT refers to the time taken to respond to a single stimulus with a single response, while CRT involves the time taken to respond to a stimulus when a decision between different possible responses is required (Schneider & McGrew, 2012).

RT evaluation is utilized across diverse fields, including cognitive and clinical psychology, sports science, neuroscience, and aging research, by various tests. However, no single test is universally recognized as the gold standard (Janicijevic & Garcia-Ramos, 2022). While some tests are designed to assess RT in a specific field or for particular skills (e.g., RT test for athletes in specific sports), most traditional tests are administered on computers, requiring participants to press a button in response to an image on a screen (Janicijevic & Garcia-Ramos, 2022). Although methodologies and protocols vary, most tests evaluate RT in controlled and artificial laboratory settings, raising concerns about their ecological validity.

Virtual reality (VR) systems offer a promising solution to this limitation by providing higher ecological validity through the creation of more lifelike environments and stimuli (Neguţ et al., 2016a; Parsons, 2015). VR’s ability to simulate real-life situations and create a strong sense of presence is expressed in its significant potential for evaluating and training RT in athletes (Faure et al., 2020). This potential could be similarly impactful in other areas, such as cognitive assessments.

Moreover, VR testing offers several advantages over traditional computerized testing. These include assessing RT in complex tasks, utilizing stimuli with diverse spatial and dynamic characteristics, and measuring various components of RT (e.g., decision time and MT) along with related factors such as eye-tracking and movement kinematics.

Despite the potential of VR for RT assessments and the growing interest in its use for cognitive evaluations, research on VR-based RT tests is still in its early stages. Several studies have investigated whether RT tests in VR yield results comparable to those obtained from traditional computerized tests. For example, Vahle et al. (2021) found no significant differences between RTs measured in a computer-based test and those measured in an identical test presented on a computer screen in a lifelike VR environment. Additionally, they demonstrated that VR tests can reflect the overall decline in performance associated with age. Similarly, two additional studies comparing RTs between traditional tests and parallel RT tests in mixed martial arts fighters concluded that VR-based RT tests are as relevant and reliable as their traditional equivalents (Langer et al., 2023; Polechoński & Langer, 2022).

Additional support for the application of VR in RT assessments is provided in the study by Campo-Prieto et al. (2023), in which researchers identified correlations between RTs measured in VR and other measures of functionality and cognitive impairment, suggesting VR’s potential for fall risk prediction in Parkinson’s disease patients.

While these studies suggest the applicability of VR for RT assessments, more research is required to substantiate these findings. In addition to validating VR as a feasible alternative to traditional RT tests, there is a need to develop novel tests that harness the unique capabilities of VR in RT research. For instance, Wang et al. (2021) developed a new VR-based procedure that assesses RT during a hand-reaching task. Unlike traditional computer tests, this VR test evaluated RT in settings that more closely resemble everyday life, using three-dimensional stimuli presented in various spatial locations and providing additional measures related to arm movements (e.g., distance, time, speed). However, this study only begins to explore the potential of VR for RT assessment, and further research is necessary to investigate the broader possibilities that VR technology offers.

In the current study, we aimed to further investigate the correlations and differences between RT performance in VR and on a computer by validating a novel VR-based RT test against a traditional computerized RT test. Additionally, we aimed to provide an initial exploration of VR’s potential for assessing RT in more dynamic and lifelike tasks. To this end, we have developed a new RT test in VR that includes:

1. An initial phase comparable to a traditional computerized RT test, and
2. A second phase with additional tasks that exploit the unique capabilities of VR, comprising reaching out to touch static and dynamic three-dimensional stimuli that appear in various locations.

These tasks were designed to remain comparable to those in the first phase while incorporating elements of everyday hand-reaching actions, such as reaching for a gear shift while driving, catching a falling object, grasping a railing to prevent a fall, or performing sport-related movements like intercepting a ball or delivering a strike. The test’s second phase may constitute an initial step toward leveraging VR to assess RT in contexts that better simulate real-world conditions.

## Methods

### Participants

The sample size calculation (see ‘statistical analysis’) yielded a requirement of 46 participants. To account for potential exclusions, an additional five participants were recruited, bringing the total number of participants to 51. Three participants were later excluded due to outlier results (SRT and CRT values exceeding two standard deviations above the group mean). This left 48 participants (26 men and 22 women) for the final analysis, with a mean age of 33.70 ± 9.16 years.

Participants were recruited through advertisements on social media and across the university. Exclusion criteria included uncorrected visual impairments, cognitive impairments (Montreal Cognitive Assessment score < 26), chronic depression, or acute illness. The study was approved by the university’s ethics committee, and all the participants signed an informed consent form before taking part in the experiment.

### Procedure

Participants attended a single laboratory examination session. Upon arrival, they first completed the Montreal Cognitive Assessment and a brief social and demographic questionnaire. Following this initial assessment, participants engaged in two RT tests: a computerized RT test (COM-RT) and an RT test in VR (VR-RT). The sequence of these tests was randomized; thus, some participants undertook the COM-RT test first, while others began with the VR-RT test. Following the VR-RT test, participants were asked to provide feedback on their experience and report any discomfort, including potential symptoms of simulator sickness.

Both RT tests were conducted in controlled environments with consistent lighting and temperature, and in both, the participants used their dominant hand for the tasks.

### Computer reaction time test

The COM-RT test was implemented in testable software (Eerola et al., 2021; Rezlescu et al., 2020) and had a duration of approximately four minutes. It was administered using an ASUS Vivobook S Flip TN3604YA laptop running Windows 11 Home, equipped with an AMD Ryzen 7 7730U processor (8 cores, 16 logical processors, base frequency 2.0 GHz), 16 GB of RAM, and integrated AMD Radeon Graphics. The laptop was connected via HDMI to a 24-inch Philips 245E1S/00 IPS LCD monitor with a resolution of 2560 × 1440 and a 75-Hz refresh rate. The laptop lid was closed during the task to ensure participants viewed only the external screen.

During the test, visual stimuli were presented on the monitor, and participants sat in front of the screen and were instructed to focus on a fixation point at its center (a black plus sign on a white background). They responded using a standard Dell USB-wired keyboard. Specifically, they were asked to press the space bar as quickly as possible when a square stimulus (4.4 cm per side) appeared, overlaid on the fixation point.

**Fig. 1:**
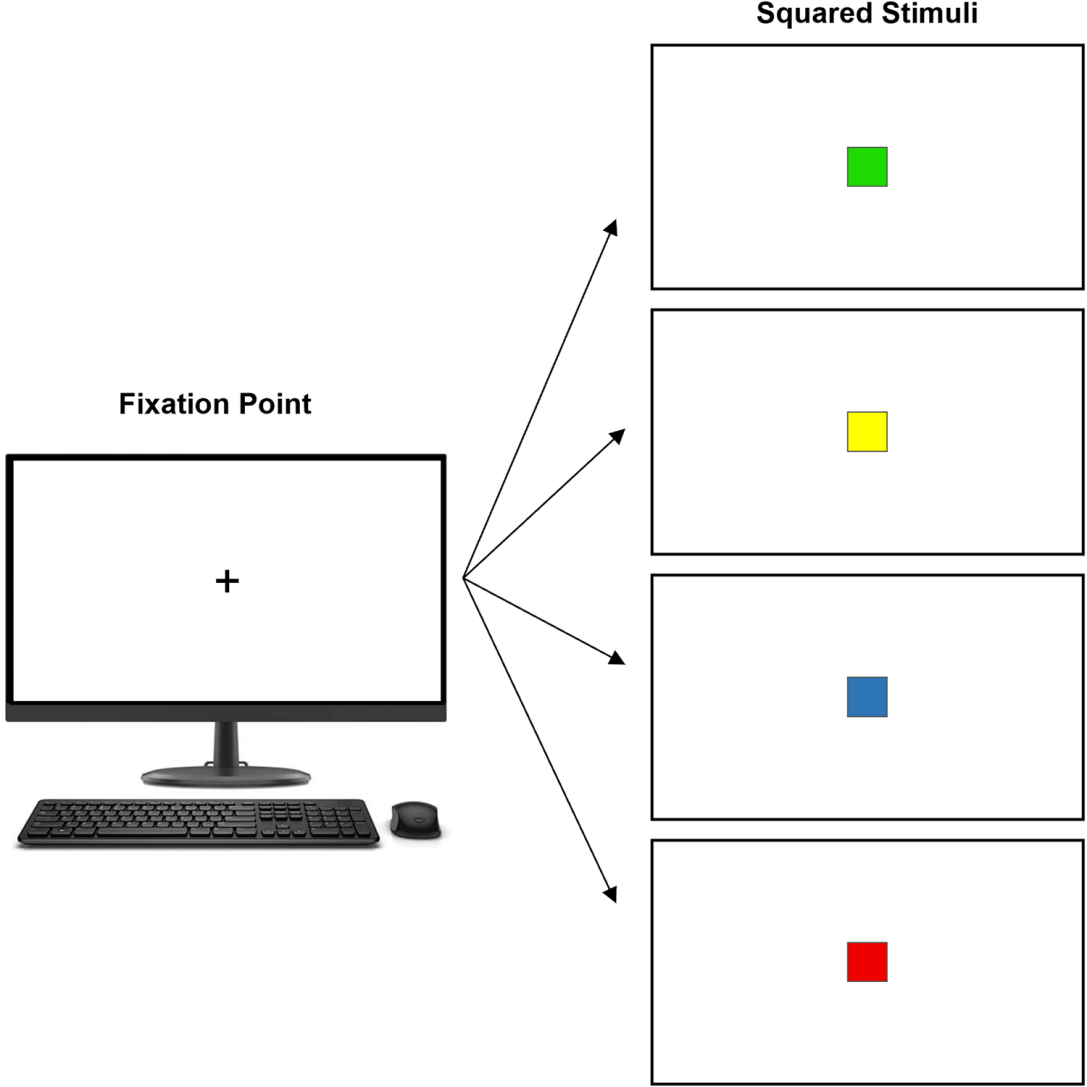
An illustration of the computer reaction time test. Participants fixated on a black plus sign at the center of a computer screen and pressed the space bar as quickly as possible when a square stimulus appeared. Initially, they responded to squares of all four colors to assess simple reaction time. Subsequently, they responded only to green and yellow stimuli and withheld responses to the other two, evaluating choice reaction time

The squares could appear in one of the four colors: yellow, green, blue, or red. The initial phase of the test assessed SRT, in which participants responded to squares of all colors (four types of go stimuli). The subsequent phase assessed CRT, where participants were instructed to respond only to two specific colors of squares and to withhold responses to the other two colors. This phase involved two types of go stimuli (green and yellow) and two types of no-go stimuli (blue and red) (Fig. 1).

Each phase included four training trials, followed by 20 trials for each stimulus type (go or no-go). Throughout the test, stimuli were presented in a random order, with an equal proportion of stimuli for each color (25%). All stimuli were displayed for 260 milliseconds (ms), with inter-stimulus intervals randomly varied between 1000 and 2000 ms.

The test protocol was primarily adapted from previously established protocols utilized in various studies involving different populations, including athletes from different disciplines (Berchicci et al., 2012; Bianco et al., 2017; Di Russo et al., 2010; Perri et al., 2014; Taddei et al., 2012). The number of repetitions employed in the current study aligns with protocols from prior research (Fontani et al., 2006; Kida et al., 2005; Nakamoto & Mori, 2008; Wang et al., 2005), and is consistent with findings suggesting that a large number of repetitions is unnecessary for accurate RT assessment (Miller, 2024). The test protocol file and accompanying materials are available in the OSF repository and can be administered using the freely available Testable software.

### Virtual reality reaction time test

The new VR-RT test was implemented on a Meta Quest Pro headset. The test had a duration of approximately 15 min and combined assessments of RTs and dynamic spatial abilities. Participants were seated on a chair without armrests and held a controller in their dominant hand. Through the headset, they viewed a virtual examination room and were required to respond as quickly as possible to stimuli during various tasks.

**Fig. 2:**
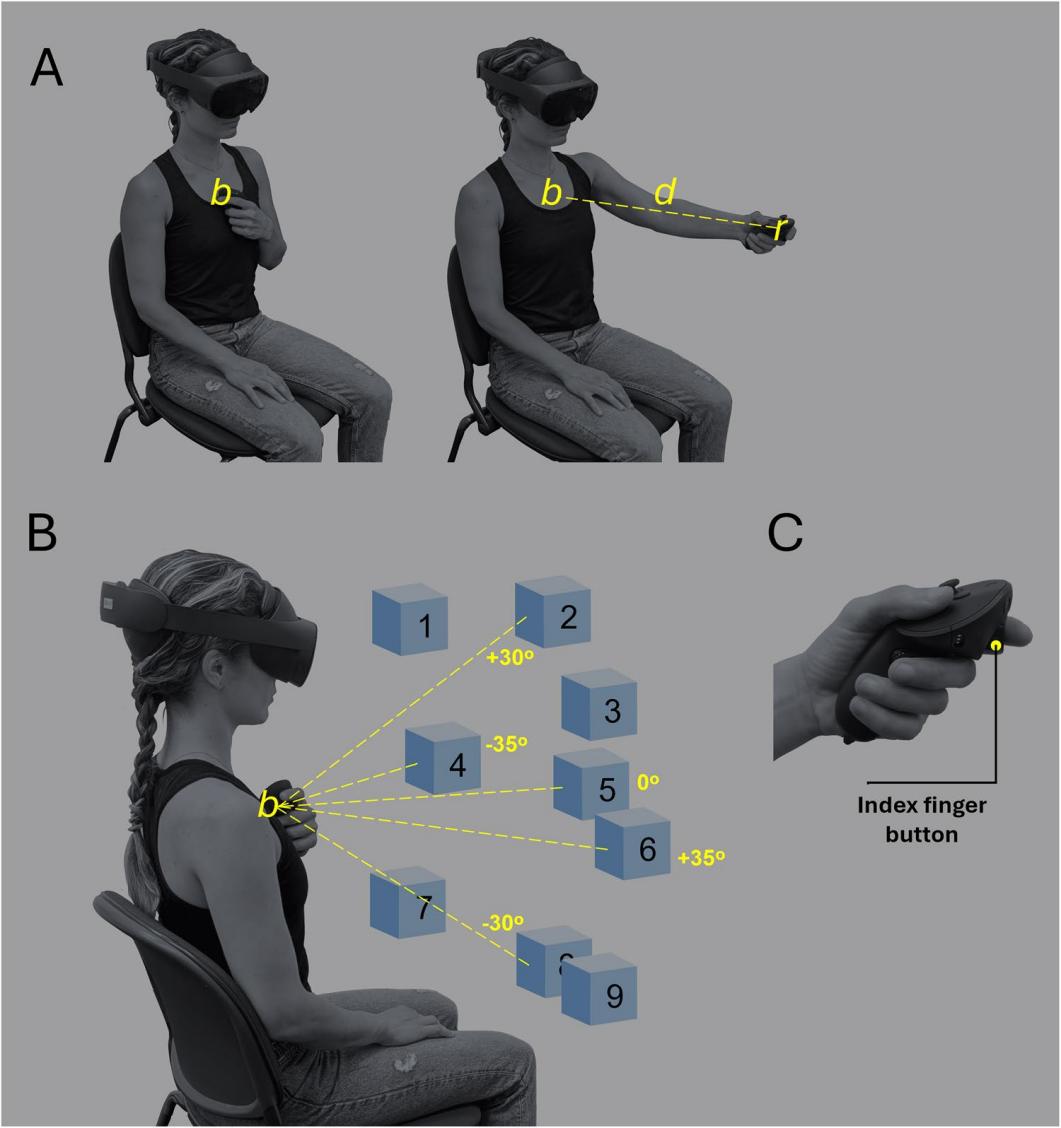
Illustration of the virtual reality reaction time test. **A** Test calibration involved determining the base (*b*) and reach (*r*) locations, and the linear distance between them (*d*). *Location r* was established with the hand extended at shoulder level, and *location b* with the controller against the sternum. **B** Nine numbered locations were defined, all at *distance d* from *location b*, and within the participant’s field of vision. *Location 5* was aligned with *location b* on both the vertical and horizontal axes, while the other locations were distributed at 30-degree angles higher or lower, and 35 degrees to the right or left (cubes 1–9). **C** Participants held a controller and used the index finger button

The test began with a brief calibration to tailor the test to each participant’s body measurements. Two landmarks were recorded: *location r* was defined as the coordinates of the controller when the participants extended their dominant hand forward at shoulder level, and *location b* corresponded to the coordinates when the controller was held against the sternum. Finally, *distance d* was calculated as the straight-line distance between *location r* and *location b* (Fig. 2A).

Based on this calibration, nine numbered locations were established around the participants. All nine locations were at the ends of invisible straight lines originating at *location b* and extending a distance equivalent to *distance d* (all at the same distance from *location b*). *Location 5* – the central location – was aligned with *location b* on the vertical and horizontal axes, and the other eight locations were distributed at 30-degree angles higher or lower, and 35 degrees to the right or left (Fig. 2B). These locations were selected to be as distinct as possible while remaining within the participant’s reach and their field of view when looking in the direction of *location* *5*.

A fixation point, in the shape of a black plus sign, was presented at *location 5* throughout the test. That is, for each participant, the fixation point was placed directly in front of the chest, at the level of the sternum, and slightly closer than the participant’s maximum reach. The stimuli presented during the test were 3D cubes with 10-cm edges, which could appear in one of four colors (yellow, green, blue, or red). When a cube appeared at a specific location (e.g., *location 3*), its front face was positioned so that its center aligned precisely with that location (i.e., at the end of the invisible line).

Following calibration, the first phase of the test began, mirroring the protocol of the COM-RT test (i.e., stimuli colors, repetitions, etc.). The minimal adaptations made for compatibility with the VR system included displaying the fixation point suspended in the air, presenting stimuli as 3D cubes, and using a button on the controller (Fig. 2C) instead of a keyboard button. The tasks in this phase were called *Press tasks*.

The second phase of the test was built upon the first, incorporating more lifelike tasks and additional performance metrics. In this phase, participants were instructed to hold the controller within a base position defined by a 0.028-m radius sphere centered around *location b*. This base sphere was strategically chosen to facilitate an easy return to position without the need for visual cues. Upon the appearance of a stimulus, participants were required to quickly reach and touch it with the controller before returning to the base position. If the controller was moved outside the base without a stimulus present, the test paused, and instructions were given to return the controller to the base. To provide sufficient time for participants to reach the stimuli throughout this stage, the cubes were displayed for 2000 ms, with inter-stimulus intervals ranging from 3000 to 4500 ms, varying randomly across trials. The other components of the protocol, including the colors of the stimuli and their random appearance, remained consistent with the COM-RT test protocol, unless otherwise noted.

This phase consisted of three distinct SRT tasks:

1. *Center task* – cubes appeared over the fixation point in *location 5*, as in the initial phase. This task included two training trials and 20 test trials;
2. *Spatial task* – cubes appeared randomly at one of the nine predefined locations. This task included two training trials and 18 test trials. In the 18 test trials, two stimuli were presented at each of the nine locations – one in a color previously used as a go stimulus (yellow or green) and the other in a color previously used as a no-go stimulus (blue or red);
3. *Dynamic task* – cubes appeared unexpectedly in one of the nine locations, while moving randomly toward one of the other eight locations. The movement speed was set at 0.6 meters per second (m/s) for the first nine repetitions, 0.75 m/s for the subsequent nine repetitions, and 0.9 m/s for the final nine repetitions. This task included two training trials and 27 test trials, with three stimuli appearing in each of the nine locations.

The test code and accompanying materials are available in the OSF repository and can be administered using Unity Editor version 2021.3.16f1.

### Measurement and calculation of reaction time and kinematics

For each task, both median and average RTs were calculated from all valid repetitions (see cutoff values below). Researchers commonly use these two measures of central tendency to describe RT; each has a different nature, and the superiority of each of them is debated (e.g., median is less influenced by outliers, whereas average is more prone to being skewed in their direction) (Ratcliff, 1993; Whelan, 2008). In the current study, correlations were examined for both median and average RTs, to establish the validation for both measures and support the use of each of them in future analyses. Outliers were carefully addressed by selecting RT thresholds between 100 and 1000 ms to exclude premature responses due to guessing and responses related to the appearance of the next stimulus (Miller, 2023; Ratcliff, 1993; Whelan, 2008).

The difference between RTs in the CRT task and the SRT (RT-Diff) was calculated as CRT minus SRT, both for the median values and the average values. In addition, the intra-individual variability of RTs was assessed using the intra-individual coefficient of variation (ICV), calculated as the standard deviation of RT divided by the mean RT. Furthermore, additional metrics were collected, including anticipations (the percentage of trials with RTs under 100 ms, out of all go stimuli trials within a task), omissions (the percentage of trials missed out of all go stimuli trials within a task, including responses exceeding 1000 ms), and false alarms (the percentage of unintentional responses, out of all no-go stimuli trials within a task).

In the VR-RT tasks involving reaching for stimuli, RT was measured from the moment the stimuli appeared until the moment the controller left the base. In these tasks, MT and movement velocity (MV) were measured as well. MT was defined as the time from the moment the controller left the base until it touched the cube, measured in ms, while MV was calculated as the movement displacement divided by MT, measured in m/s. Movement displacement refers to the gap between the location of the controller when the cube appeared and its location when the cube disappeared (i.e., when the participant touched it), with a subtraction of 0.028 m. This subtraction was applied to adjust the movement displacement to MT, which was measured only after the controller left the base sphere (i.e., after it moved an average displacement of approximately 0.028 m).

An initial examination of the MT data revealed a right-skewed distribution, with outliers characterized by excessively long durations (three to six times greater than the participant’s average MT of all other trials within the task). These outliers represented unusual cases, such as when the participant missed the cube with the controller and had to make an additional full movement to touch it. Therefore, a robust measures approach for handling outliers was employed, using an upper cut-off value of 600 ms (Miller, 2023; Ratcliff, 1993; Whelan, 2008). Applying this cut-off excluded only 2.6% of the MTs from the total sample, with 81.5% of these excluded values characterized by excessively long durations (Supplementary Table 1).

For each participant, median and average values were recorded for both MT and MV. Additionally, ICV was calculated for both measures. Two additional measures were recorded for the MT. One was referred to as over-600 (the percentage of trials with MTs longer than 600 ms, out of all trials within a task), and the other as movement-omissions (M-omissions), representing the percentage of trials where the participant failed to touch the stimulus before it disappeared despite having valid RTs, out of all trials within a task.

### Statistical analyses

The sample size was calculated using G*Power 3.1 (Faul et al., 2007, 2009). An a priori power analysis was conducted for a two-tailed correlation analysis, which was the primary statistical test of interest in this study. To detect a medium effect size (*r* = 0.4) with a power of 0.80 and an alpha level of 0.05, the analysis indicated a required sample size of 46 participants.

The statistical analyses were conducted using SPSS Statistics 26 (IBM Corporation, Armonk, NY, USA). Normality was assessed using histograms and the Kolmogorov–Smirnov test. Descriptive statistics were used to characterize the sample in terms of participant demographics and RT measures.

Validation of the novel VR-RT test in comparison to the traditional COM-RT test was conducted using Pearson correlation coefficients for RT and RT-Diff measures, to assess the strength of linear associations between the two methods. Bland–Altman analyses were also performed to further evaluate agreement and detect any systematic or proportional bias between the methods. Comparisons between the tests for additional RT-related variables – including ICV, omissions, anticipations, and false alarms – were conducted using Wilcoxon signed-rank tests, as these variables were not normally distributed. A Bonferroni correction was applied to control for multiple comparisons, i.e., the seven tests (adjusted α = 0.0071).

To further explore differences between simple and complex RTs across both testing modalities, a repeated-measures ANOVA was conducted. When the assumption of sphericity was violated, as assessed by Mauchly’s test, the Greenhouse–Geisser correction was applied to adjust the degrees of freedom. Standard Bonferroni corrections were applied within the repeated-measures ANOVA to control for multiple post-hoc comparisons.

To explore differences in RT, MT, and MV across the VR-RT tasks, repeated-measures ANOVAs were conducted as described above. Comparisons between the VR-RT tasks for additional variables that were not normally distributed (ICV, omissions, anticipations, M-omissions, and over-600) were conducted using the Friedman test. To control for multiple comparisons across the seven tests, a Bonferroni correction was applied (adjusted α = 0.0071). For variables with significant Friedman test results, post hoc analyses were performed using pairwise Wilcoxon signed-rank tests. Bonferroni corrections were applied according to the number of pairwise comparisons (adjusted α = 0.0083 for comparisons across all four tasks, and adjusted α = 0.0166 for comparisons across three tasks). Additionally, Pearson’s correlations were conducted to examine the associations between VR tasks for each of the variables: RT, MT, and MV. A Bonferroni correction was applied to control for multiple comparisons across 12 correlation pairs (adjusted α = 0.0042).

## Results

### Descriptive statistics, correlations, and comparisons between the two tests

Detailed data about subjects' characteristics are provided (Table 1). Twenty-three participants (48%) first performed the COM-RT test and then the VR-RT test, while 25 (52%) performed the tests in the opposite order. None of the participants reported any signs of simulator sickness. RT descriptive statistics are provided (Table 2). No significant differences (adjusted α = 0.0071) were found between the COM-RT and the VR-RT tests in ICV (SRT: *p* = 0.669; CRT: *p* = 0.479), omission rates (SRT: *p* = 0.644; CRT: *p* = 0.160), anticipation rates (SRT: *p* = 0.078; CRT: *p* = 1.000), or false alarm rates (CRT: *p* = 0.078).

**Table 1:** Subjects’ characteristics

Variable		Value *n* = 48
Age (years)		33.70 ± 9.16
Sex	Male	26 (54%)
	Female	22 (46%)
Dominant hand	Right	40 (83%)
	Left	8 (17%)
Use of glasses or contacts	None	28 (58%)
	Nearsighted correction	6 (13%)
	Farsighted correction	11 (23%)
	Both	3 (6%)
Attention deficit disorder	Yes	4 (8%)
	No	44 (92%)
Education (years)		16 [12–27]
MoCA (total score)		28 [26–30]
General computer experience (1–5)		5 [3–5]
Computer game experience (1–5)		3 [1–5]
Virtual reality experience	Never	16 (33%)
	Once	19 (40%)
	Twice or more	13 (27%)
Physical activity classification (Tier)	Sedentary	36 (75%)
	Recreationally active	9 (19%)
	Trained/developmental	0 (0%)
	Highly trained/national level	2 (4%)
	Elite/international level	0 (0%)
	World-class	1 (2%)

Number (%) is presented for nominal variables, median [min-max] is presented for ordinal variables and quantitative variables that are not normally distributed, and mean ± SD is presented for quantitative variables that are normally distributed. MoCA Montreal cognitive assessment

**Table 2:** Descriptive statistics of reaction time variables in computer and virtual reality tests

Category	Variable	Computer (x̄ ± SD)	Virtual reality (x̄ ± SD)
		*n* = 48	*n* = 48
Simple reaction time	SRT^M^ (ms)	239.42 ± 19.07	271.86 ± 20.76
	SRT^A^ (ms)	244.85 ± 21.71	280.49 ± 24.18
	ICV (ms)	0.14 ± 0.05	0.14 ± 0.09
	Omissions (%)	0.01 ± 0.03	0.01 ± 0.02
	Anticipation (%)	0.01 ± 0.03	0.01 ± 0.02
Choice reaction time	CRT^M^ (ms)	360.91 ± 39.84	386.38 ± 49.80
	CRT^A^ (ms)	369.55 ± 38.84	396.26 ± 47.14
	ICV (ms)	0.17 ± 0.04	0.18 ± 0.04
	Omissions (%)	0 ± 0.01	0.01 ± 0.02
	Anticipation (%)	0 ± 0	0 ± 0
	False Alarms (%)	0.07 ± 0.08	0.09 ± 0.08
Simple-choice reaction time difference	RT-Diff^M^ (ms)	121.49 ± 38.40	114.51 ± 46.70
	RT-Diff^A^ (ms)	124.70 ± 38.39	115.77 ± 47.17

Quantitative variables are represented by mean ± standard deviation. RT reaction time; SRT^M^ median simple RT; SRT^A^ average simple RT; CRT^M^ median choice RT; SRT^A^ average choice RT; RT-Diff^M^ median RT difference (median choice RT minus median simple RT); RT-Diff^A^ average RT difference (average choice RT minus average simple RT); ICV intra-individual coefficient of variation (standard deviation of the measure divided by its mean); anticipations (the percentage of trials with RTs under 100 ms, out of all go stimuli trials within a task), omissions (the percentage of trials missed out of all go stimuli trials within a task, including responses exceeding 1000 ms); false alarms (the percentage of unintentional responses, out of all no-go stimuli trials within a task)

**Fig. 3:**
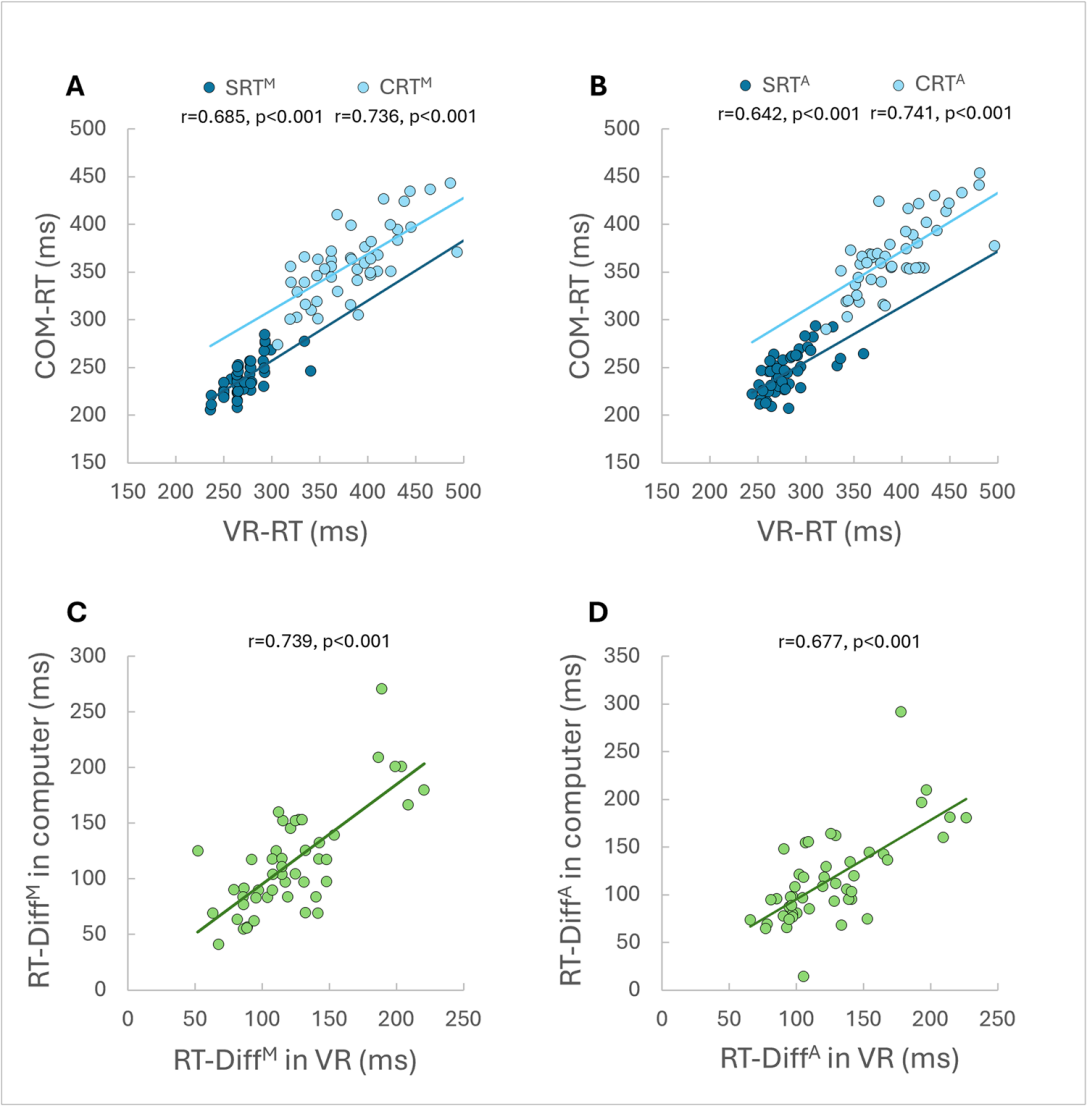
Correlations between reaction times (RTs) in computerized and virtual reality tests. Significant moderate-to-strong linear correlations were found between the tests. COM-RT computer RT; VR-RT virtual reality RT; SRT^M^ median simple RT; SRT^A^ average simple RT; CRT^M^ median choice RT; CRT^A^ average choice RT; RT-Diff^M^ median RT difference (subtraction of median simple RT from median choice RT); RT-Diff^A^ average RT difference (subtraction of average simple RT from average choice RT)

**Fig. 4:**
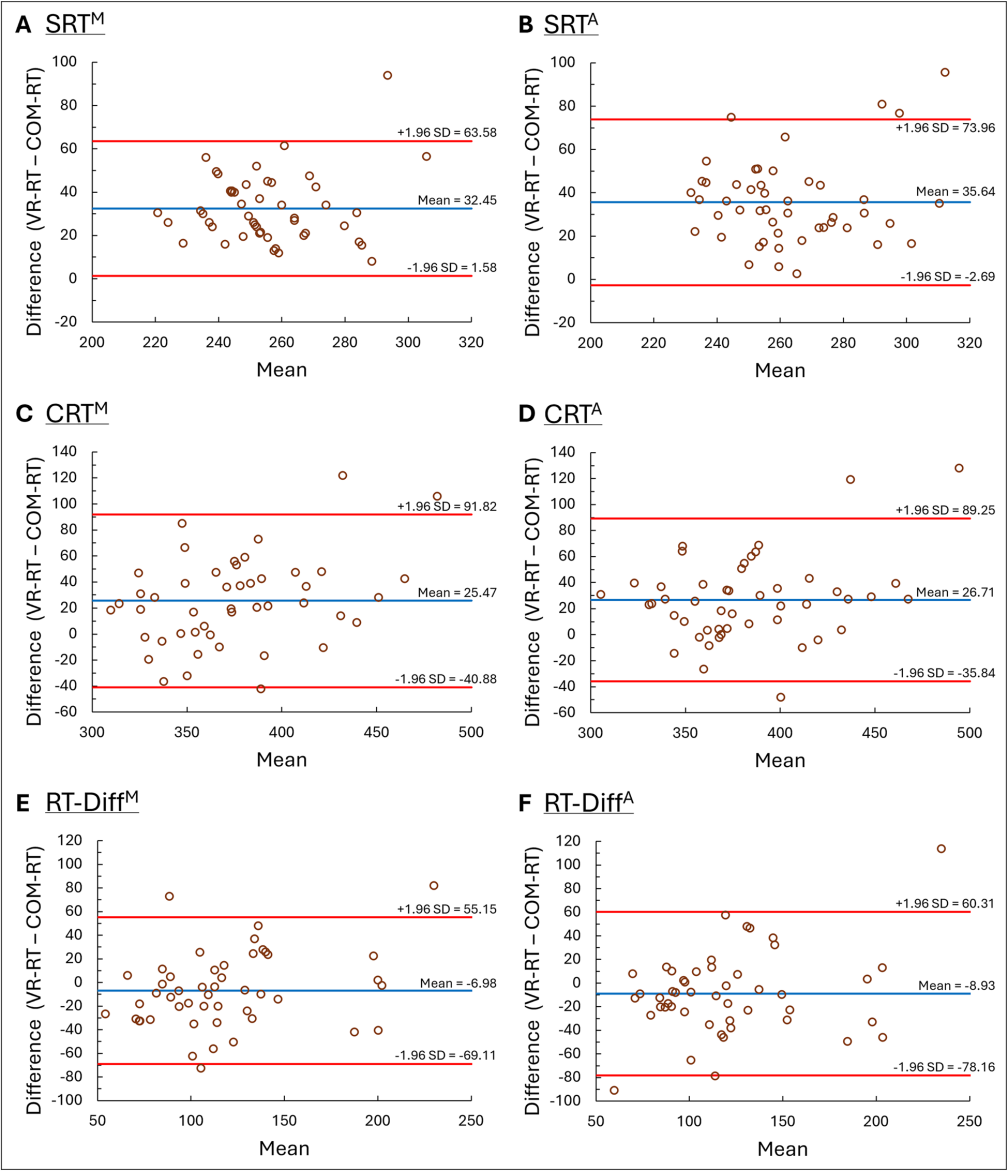
Bland–Altman analysis comparing reaction times (RT) from computerized and virtual reality (VR) tests. A consistent systematic bias was observed, with VR-based RTs exceeding computer-based RTs across all conditions. No proportional bias was detected, yet the analysis revealed wide limits of agreement. COM-RT computer RT; VR-RT RT in VR; SRT^M^ median simple RT; SRT^A^ average simple RT; CRT^M^ median choice RT; CRT^A^ average choice RT; RT-Diff^M^ median RT difference (subtraction of median simple RT from median choice RT); RT-Diff^A^ average RT difference (subtraction of average simple RT from average choice RT)

Significant moderate-to-strong linear correlations were found between the two tests for: SRT, CRT, and RT-Diff. The correlations were found for both median RTs and average RTs (Fig. 3). Bland–Altman analysis revealed a consistent systematic bias across all conditions, with VR-based RTs exceeding computer-based RTs by approximately 26–36 ms for SRT and CRT, and by 7–9 ms for RT-Diff. No clear evidence of proportional bias was found, as the differences did not vary systematically with the magnitude of the measurements. However, wide limits of agreement were observed in all comparisons (Fig. 4).

**Fig. 5:**
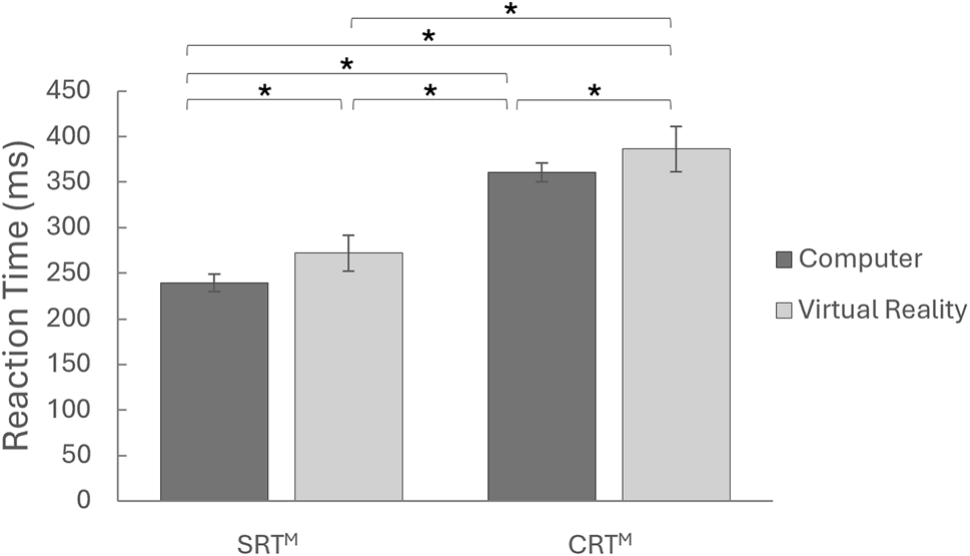
Comparison between simple and choice reaction times (RTs) in the computerized and virtual reality tests. Significant differences were found between RT conditions (*p* < 0.001), with each of the conditions significantly different from all other three conditions (*p* < 0.001). SRT^M^ median simple RT; CRT^M^ median choice RT; Error bars represent standard deviation; * *p* < 0.001. *Note*: average RT yielded similar results

A repeated-measures ANOVA comparing SRTs and CRTs across both testing modalities revealed a significant main effect of task on RT, *F*(1.73, 81.48) = 311.64, *p* < 0.001, partial η^2^ = 0.869. Bonferroni-adjusted post hoc comparisons indicated significant differences between all four tasks (*p* < 0.001). Specifically, SRTs were significantly shorter than CRTs in both the COM-RT and the VR-RT tests. In addition, both variables were significantly shorter in the COM-RT test compared with the VR-RT test (Fig. 5).

As the results for median and average RTs were similar across all analyses from this point onward, only median RTs are presented in the figures. The same approach is applied to MT and MV results.

### Reaction time and kinematic data in virtual reality

Descriptive statistics of the data acquired during the second phase of the VR-RT test are provided (Table 3). Significant moderate to strong correlations were found between several VR tasks for RT, MT, and MV (based on the adjusted α = 0.0041). RT in the *Press* task showed a significant moderate linear correlation with the *Center* task (*r* = 0.486, *p* < 0.001). Moreover, all correlations between the *Center*, *Press*, and *Dynamic* tasks for RT, MT and MV were significant, with coefficients ranging from moderate to strong (*r* = 0.525 to *r* = 0.902, *p* < 0.001). Full correlation matrices are provided in Supplementary Table 2.

**Table 3:** Descriptive statistics of tasks involving reaching for stimuli

Category	Variable	Center (x̄ ± SD) *n* = 48	Spatial (x̄ ± SD) *n* = 48	Dynamic (x̄ ± SD) *n* = 48
Simple reaction time	SRT^M^ (ms)	341.38 ± 43.60	386.00 ± 44.18	344.77 ± 35.39
	SRT^A^ (ms)	352.24 ± 45.24	393.56 ± 46.07	351.47 ± 35.09
	ICV (ms)	0.16 ± 0.07	0.12 ± 0.05	0.12 ± 0.05
	Omissions (%)	0.01 ± 0.02	0 ± 0.02	0 ± 0.01
	Anticipation (%)	0 ± 0.01	0 ± 0.01	0 ± 0.01
Movement time	MT^M^ (ms)	187.41 ± 56.70	191.30 ± 56.60	147.06 ± 45.53
	MT^A^ (ms)	191.66 ± 56.58	198.66 ± 55.13	157.72 ± 45.67
	ICV (ms)	0.17 ± 0.08	0.23 ± 0.14	0.33 ± 0.16
	M-Omissions (%)	0.01 ± 0.04	0.01 ± 0.03	0.10 ± 0.10
	Over-600 (%)	0.01 ± 0.02	0.06 ± 0.06	0.01 ± 0.02
Movement velocity	MV^M^ (m/s)	2.96 ± 0.96	2.90 ± 0.92	3.62 ± 0.95 *
	MV^A^ (m/s)	2.95 ± 0.95	2.90 ± 0.90	3.64 ± 0.93 *
	ICV (m/s)	0.15 ± 0.04	0.20 ± 0.07	0.23 ± 0.07 *

Quantitative variables are represented by mean ± standard deviation. SRT^M^ median simple reaction time; SRT^A^ average simple reaction time; ICV intra-individual coefficient of variation (standard deviation of the measure divided by its mean); Anticipations (the percentage of trials with RTs under 100 ms, out of all go stimuli trials within a task); Omissions (the percentage of trials missed out of all go stimuli trials within a task, including responses exceeding 1000 ms); MT^M^ median movement time; MT^A^ average movement time; M-Omissions movement time omissions (representing the percentage of trials where the participant failed to touch the stimulus before it disappeared despite having valid RTs, out of all trials within a task); Over-600 (the percentage of trials with movement time longer than 600 ms, out of all trials within a task); MV^M^ median movement velocity; MV^A^ average movement velocity. *n =47 due to malfunction and missing data from one participant

**Fig. 6:**
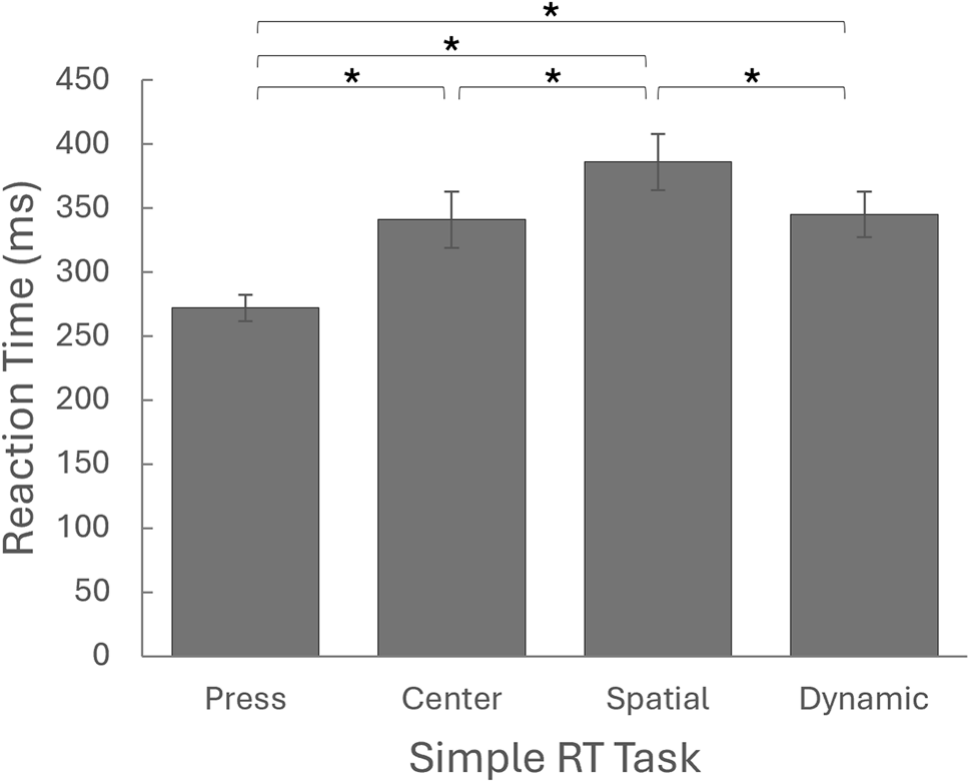
Comparison of reaction time (RT) between various tasks in virtual reality. Significant differences in median simple RT were found between the different tasks in the virtual reality test (*p* < 0.001). Each RT condition was significantly different from each of the other conditions (*p* < 0.001), except for the *Center* and *Dynamic* conditions, which were similar to each other (*p* =1.000). Error bars represent standard deviation; * *p* < 0.001. *Note*: average RT yielded similar results

To further examine task differences in RT, a repeated-measures ANOVA was conducted, revealing a significant main effect of task on RT, *F*(2.41,113.09) = 163.64, *p* < 0.001, partial η^2^ = 0.777. Bonferroni-adjusted post hoc comparisons indicated that RT was shortest in the *Press* task, increased in the *Center* task, and was longest in the *Spatial* task (*p* < 0.001). Notably, the RT in the *Dynamic* task did not differ significantly from the *Center* task (*p* =1.000), but was significantly shorter than in the *Spatial* task and longer than in the *Press task* (*p* < 0.001) (Fig. 6). Additionally, analyses of RT-related variables revealed no significant differences between tasks in ICV, omission rates, or anticipation rates (*p* = 0.031, *p* = 0.172, and *p* = 0.178, respectively), as all values exceeded the adjusted significance threshold (α = 0.0071).

**Fig. 7:**
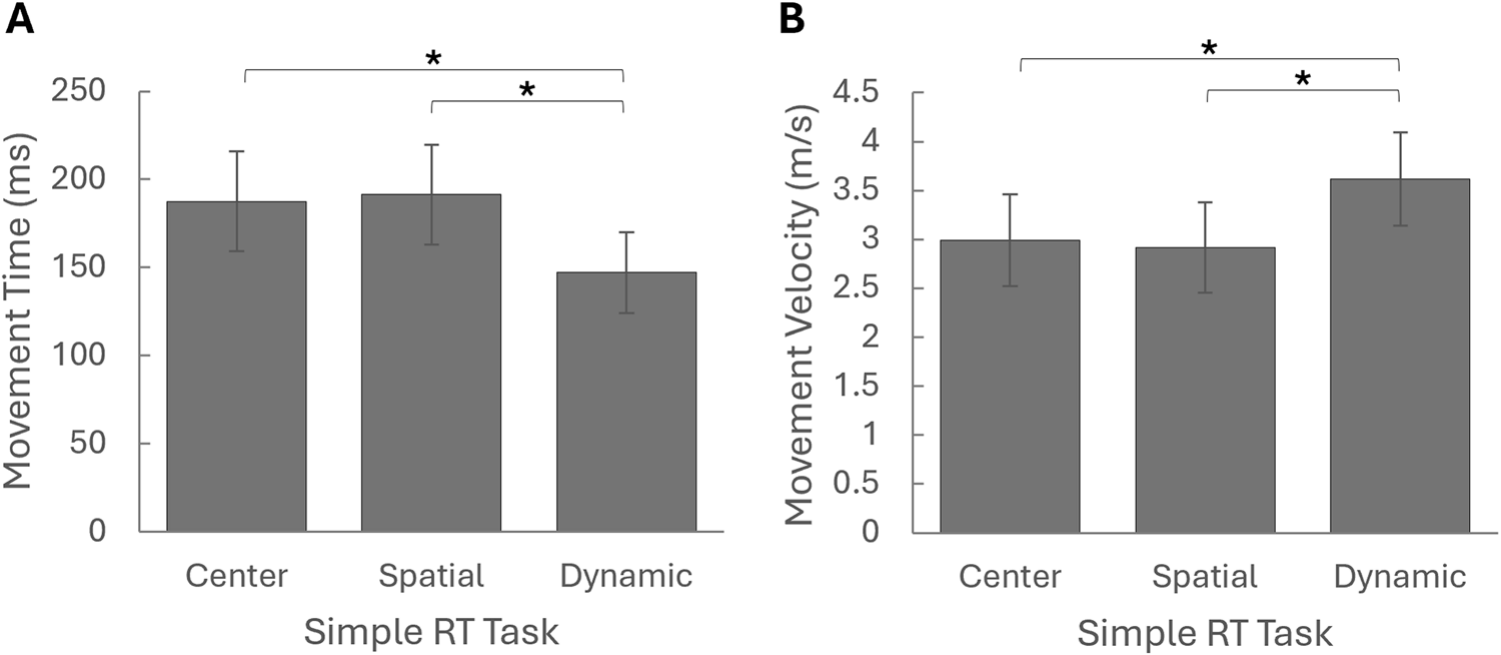
Comparison of movement time and velocity between different tasks in virtual reality. Significant differences were found for median movement time **A** and median movement velocity **B** between the tasks (*p* < 0.001). Post hoc analysis revealed significant differences between the *Dynamic task* and the two other tasks (*p* < 0.001), with no significant differences between the Center and the Spatial tasks (*p* =1.000 for median movement time, and *p* = 0.677 for median movement velocity). *Error bars* represent standard deviation; * *p* < 0.001. *Note*: average movement time and velocity yielded similar results

Regarding task differences in kinematic metrics, repeated-measures ANOVAs revealed significant differences in MT and MV across the different VR-RT tasks involving reaching for stimuli. For MT, *F*(2,94) = 41.32, *p* < 0.001, partial η^2^ = 0.468. For MV, *F*(1.70, 78.32) = 62.02, *p* < 0.001, partial η^2^ = 0.574. Bonferroni-adjusted post hoc comparisons revealed that in the *Dynamic* task, MT was significantly shorter and MV was significantly higher than in the *Center* and *Spatial* tasks (*p* < 0.001). No significant differences were found between the *Center* and *Spatial* tasks (MT: *p* =1.000; MV: *p* = 0.677) (Fig. 7).

Further analysis revealed that ICV differed significantly across tasks for both MT and MV (*p* < 0.001; adjusted α = 0.0071). Post hoc analyses revealed that ICV was lowest in the *Center* task, higher in the *Spatial* task, and highest in the *Dynamic* task (*p* ≤ 0.009; adjusted α = 0.0166). Additionally, significant differences were found between tasks in the rates of M-omissions and over-600 (*p* < 0.001; adjusted α = 0.0071). Post hoc analyses (adjusted α = 0.0166) revealed that the M-omission rate was significantly higher in the *Dynamic* task compared to both the *Center* and *Spatial* tasks (*p* < 0.001), while no significant difference was observed between the *Center* and *Spatial* tasks (*p* = 0.719). On the other hand, the over-600 rate was significantly higher in the *Spatial* task than in both the *Center* and *Dynamic* tasks (*p* < 0.001), with no significant difference between the *Center* and *Dynamic* tasks (*p* = 0.242).

## Discussion

The primary aim of this study was to investigate the correlations between RT performance in the novel VR-RT test and the COM-RT test. The results demonstrated significant moderate-to-strong linear correlations for SRT, CRT, and RT-Diff. However, a systematic bias was identified, with VR-RT values being consistently longer than those recorded in the COM-RT test, accompanied by wide limits of agreement. These findings indicate that while the VR-RT test is a valid assessment tool, it is not directly interchangeable with the COM-RT test, and results should be interpreted within their respective testing contexts.

The study also aimed to provide an initial exploration of VR’s potential for assessing RT in more dynamic and lifelike scenarios. To this end, additional tasks with varying spatial and dynamic complexities were developed. Analyses revealed significant moderate to strong linear correlations across the different VR tasks, as well as significant differences between tasks in RT, MT, and MV. These results suggest that, although the more complex tasks build upon the simpler ones, they may capture additional aspects of RT assessment. Therefore, introducing dynamic and spatial complexity in VR tasks may engage a broader range of cognitive and motor processes, and support the potential of VR for more complex RT assessments while emphasizing the need for further refinement and development of such tools.

### Validation of the novel virtual reality test

The significant moderate-to-strong correlations between the COM-RT and the VR-RT tests for SRT, CRT, and RT-Diff indicate that the VR-RT test captures similar underlying cognitive processes as the traditional COM-RT test. These correlations are crucial, suggesting that the VR-RT test is a valid tool for measuring RT, while also offering the additional benefits of an immersive and interactive testing environment. This validation is supported by the consistent correlations for both simple and choice RTs, and for both average and median measures. Furthermore, since RT-Diff reflects the cognitive load imposed by the CRT task for each participant, the additional correlations found for this measure further reinforce the VR-RT test's validity. The absence of significant differences in ICV, omissions, anticipations, and false alarms between the two tests also supports the test's validity, indicating comparable attentional consistency and inhibitory control across systems.

The results from the current study correspond with Langer et al. (2023) and Polechoński & Langer (2022), who reported similar correlations between RT tasks in VR and different RT tasks on a computer. Langer et al. (2023) also compared a VR-based ruler drop task with its traditional real-world version. Interestingly, despite the moderate-to-strong correlations they found between the VR test and the computer test, the correlation between the VR test and its traditional real-life version was only low to moderate. The researchers explained this finding by the influence of human factors in the real-life version (Langer et al., 2023). Our study supports and extends these findings by demonstrating correlations in a more general population, not limited to athletes. Additionally, using two similar versions of the same RT tasks for both the computer and the VR tests in our study further clarifies the validity of RT assessment in VR.

To complement the correlation analysis, Bland–Altman analyses were conducted to assess the level of agreement between the two methods. The results revealed a consistent systematic bias, with VR-RT values being approximately 26–36 ms longer than COM-RT values across all conditions. However, no proportional bias was detected. This suggests that while the two systems are aligned in terms of the relative ranking of individuals (as shown by the correlations), the absolute RT values differ systematically. The wide limits of agreement further indicate that the two methods are not directly interchangeable, underscoring the importance of interpreting VR-based RT results within the context of the specific system used.

This systematic bias aligns with the significantly longer SRT and CRT observed in the VR-RT test compared with the COM-RT test. This discrepancy is consistent with previous findings (Langer et al., 2023; Polechoński & Langer, 2022) and may be attributed to two key factors: technical and biological. Importantly, these RT differences do not diminish the validity of the VR-RT test but emphasize the need for caution when comparing RT results across different systems, even when the tasks appear similar.

A key technical factor that may affect the comparison between VR and computerized tests is the use of different hardware and software (Langer et al., 2023; Polechoński & Langer, 2022). In our study, the VR-RT test utilized a Bluetooth-connected controller (Bluetooth 5.2), while the COM-RT test employed a wired keyboard. Nevertheless, we ensured a stable Bluetooth connection throughout the VR testing by conducting the experiment in an environment with minimal wireless interference, maintaining close proximity between controllers and the headset, and ensuring all devices were fully charged before each session.

Additionally, biological factors may have contributed to the longer RTs observed in the VR-RT test. One possible factor is hand positioning and muscle engagement, as the VR-RT test required pressing a button on a controller while the COM-RT test used a keyboard button. However, in both tests, participants used their index fingers to press the response buttons, making these motor variations subtle. Another factor could be the participants’ relative inexperience with VR, compared with their familiarity with computers. In this context, according to a recent review, VR-based assessments are more difficult in various cognitive domains, compared with paper-and-pencil or computerized assessments. This may stem from the inherent complexity of VR tasks, which allow for the simulation of real-world environments and can introduce stressors, distractors, and complex stimuli (Neguţ et al., 2016a). In the current study, although both tasks were designed to be as similar as possible, they differed in perceptual aspects related to stimulus presentation – whether on a screen or in mid-air – as well as in variations of stimulus size, distance, and dimensionality. Previous research suggests that these factors can have variable and complex effects on cognitive perception and cognitive load (Dan & Reiner, 2017; Nejati, 2021; Plewan & Rinkenauer, 2017).

Moreover, the impact of spatial cues on target detection and attention may offer another explanation. Previous studies highlight the complex effects of fixation point locations (Summers & Meese, 2009), characteristics of spatial cues, and areas of attentional focus (Benso et al., 1998; Castiello & Umilta, 1990; Huang et al., 2016) on target detection and attention fields. Based on this, the bounded screen area in our COM-RT test may have functioned as a spatial cue that facilitated attention or improved stimulus detection, thereby shortening RTs. However, further research is needed to investigate this potential effect.

In light of these technical and biological considerations, the study by Vahle et al. (2021) is particularly relevant. The researchers demonstrated that conducting the same RT test in both real-world and VR environments resulted in no significant differences in performance. In their protocol, participants used the same controller for both tests and responded to identical stimuli displayed on a computer screen, regardless of the environment. However, the extent to which the similarity in results can be attributed to technical versus biological factors remains unclear.

Finally, VR appears to be a valid tool for assessing RT. Nonetheless, further investigation into the underlying causes of the differences between VR-based and computer-based RT assessments is necessary and may yield valuable insights.

### Reaction time data in the novel virtual reality test

The second phase of the VR-RT test revealed significant variations in RT across tasks of different complexities, while no significant differences were observed between tasks in RT-related variables (ICV, omissions, and anticipations). The variation in RTs across VR-RT tasks highlights the considerable impact of motor demands on RT, as well as the spatial and dynamic nature of stimuli. For example, the shorter RT observed in the *Press *task compared with the *Center *task implies that simpler motor responses, such as pressing a button, require less cognitive processing and planning than more complex motor actions like reaching, which involve greater degrees of freedom. Prior research has shown that different motor tasks are associated with distinct brain activity patterns (Herz et al., 2012; Luft et al., 2002; Nathan et al., 2012; Pellizzer & Hedges, 2003), supporting the idea that movement complexity influences the time required to initiate it.

Furthermore, the longest RT observed in the *Spatial *task, underscoring the additional cognitive load involved in localizing stimuli in unpredictable positions and preparing the corresponding reaching movement. This aligns with the findings of Carreiro et al. (2011), who reported longer RTs when the location of stimuli on a computer screen was more unpredictable. Other studies also support the notion that spatial uncertainty regarding potential stimuli locations slows detection and results in longer RTs (Beck et al., 2014; Gros et al., 2005; Huang et al., 2016; Jensen & Munro, 1979; Pellizzer & Hedges, 2003). Additionally, the spatial distribution of potential target locations significantly influences the preparation of reaching movements (Chapman et al., 2010; Gallivan & Chapman, 2014; Ghez et al., 1997; Stewart et al., 2013), which, in turn, affects the resulting RTs (Bock & Arnold, 1992; Bock & Eversheim, 2000; Pellizzer & Hedges, 2003).

Interestingly, the RT in the *Dynamic* task was similar to that in the *Center *task and shorter than in the *Spatial* task. This is noteworthy because dynamic stimuli, which introduce motion on top of spatial uncertainty, could potentially increase the cognitive load by requiring predictive planning and more complex movement strategies. However, these findings align with previous research showing shorter RTs in response to moving stimuli compared with static stimuli (Aschersleben & Musseler, 1999; Engelken et al., 1991; Gros et al., 2005; Van Thiel et al., 2000).

The shorter RTs observed in the *Dynamic* task compared to the *Spatial* task may be attributed to inherent factors influencing RT. Some researchers have suggested that dynamic stimuli capture attention more efficiently than static stimuli, thereby reducing the processing time for RT tasks (Chastain et al., 2002; Egeth & Yantis, 1997; Skarratt et al., 2014; Washburn, 1993). However, this idea is somewhat challenged when considering the results of Carreiro et al. (2011), which suggests that the shorter RTs observed for more intense stimuli are primarily driven by sensory mechanisms in the early stages of visual processing, rather than by attentional processes. This perspective is further supported by Lange-Malecki & Treue (2012), who found that visual motion engages similar attention and decision-making processes as static visual features.

In support of this notion, previous research revealed that the visual system processes moving and stationary objects through different channels (Frassinetti et al., 2009; Manzone & Tremblay, 2023; Merchant & Georgopoulos, 2006; Noda et al., 1971; Palmer & Davis, 1981). Some studies suggest that moving stimuli are detected more efficiently (Gros et al., 1996, 2005; Van Thiel et al., 2000), while others note that these differences are embedded in early stimulus processing that transmits directly into the motor system without requiring complex cognitive processing (Aschersleben & Musseler, 1999).

Interestingly, observing visual motion seems to affect not only the processing of visual stimuli but also auditory stimuli. For example, sounds were perceived as louder when accompanied by moving visual stimuli compared with static visual stimuli (Maniglia et al., 2017). Additionally, auditory RTs were shorter when participants observed interactions between two fast-moving stimuli compared with slower ones (Manzone & Tremblay, 2023). These findings suggest that there is much to explore regarding the mechanisms behind the influence of movement on RT.

### Kinematic data in the novel virtual reality test

When examining the kinematic data for the participants’ reaching movements, we observed significant differences between the *Dynamic *task and both the *Center* and *Spatial* tasks. The significantly shorter MT and higher MV in the *Dynamic *task demonstrate that, in some cases, the nature of the task can lead to more efficient motor execution. This is consistent with findings from Van Thiel et al. (2000), where MT was shorter when reaching moving targets compared with stationary targets.

In contrast, there were no significant differences in MT or MV between the *Center* and *Spatial* tasks. This suggests that, while additional spatial demands may affect RTs, they do not necessarily influence the execution phase of the movement itself. This is reasonable, as many motor control processes related to factors of distance and velocity are often completed before movement execution (Yeom et al., 2020). Consistent with this, a previous study involving reaching to stimuli with a joystick found that, although spatial uncertainty increased RTs, it did not affect MT. This suggests that directional uncertainty impacts motor planning but not movement execution (Pellizzer & Hedges, 2003).

Another comparable example is present in the study of Gutiérrez-Dávila et al. (2013), which examined RT and kinematics in fencers during sport-specific movements. In their experiment, fencers assumed a typical fencing position and executed an attack as quickly as possible in response to a stimulus on a screen. Although the stimulus appeared static, in some trials it began to move after the fencers had already initiated their movement. While uncertainty regarding the direction of stimulus movement increased RTs in correlation with the number of possible directions, it did not affect MT, precision, or the segmental participation sequence.

That said, it is worth noting that although adding spatial demands in the current study did not appear to affect the average or median MT or MV, it did influence the variability of these measures. Specifically, ICVs for both MT and MV were higher in the *Spatial* task compared to the *Center* task. This suggests that the greater variability in stimulus locations across trials led to increased variability in motor execution, even though it did not affect central tendency measures. Furthermore, ICVs were even higher in the *Dynamic* task than in the *Spatial* task, reinforcing the idea that increased variability in stimulus position across trials influences the variability of motor execution.

Additional differences between tasks were observed in the rates of M-omissions and over-600 responses. These findings highlight the value of using more detailed kinematic measures to evaluate differences in motor performance. Nevertheless, the current study provides only an initial glimpse into the potential of VR-based kinematic analysis, and specific conclusions should be drawn with caution. Future research could further explore how task demands – such as spatial uncertainty and stimulus movement – affect kinematic variables.

### Implications

The successful validation of the VR-RT test opens new possibilities for RT assessments in more realistic VR settings. The ability of VR to simulate complex, dynamic environments allows for the creation of testing scenarios that better reflect real-world conditions, potentially leading to a more accurate representation of behavior outside the lab. This is particularly valuable in domains where sensorimotor function is critical, such as neuropsychological assessments, rehabilitation, and sports, as well as in areas involving dynamic and spatially complex tasks, such as driving and aviation.

The distinct patterns of RT, MT, and MV observed in the VR-RT test highlight the potential of VR for assessing a wider range of cognitive and motor functions than traditional RT tests. The similarity in RT between the *Center* and *Dynamic* tasks, despite the increased complexity of the latter, suggests that the VR-RT test may be useful in identifying adaptive strategies or compensatory mechanisms. Furthermore, the ability to collect kinematic indicators alongside RT measures emphasizes the advantages of the VR-RT test, and VR testing in general, for a more comprehensive evaluation of RT performance and the underlying sensorimotor processes.

## Limitations

While the present study offers validation for the novel VR-RT test, several limitations should be acknowledged. First, although the VR-RT test environment is more immersive than computer-based setups, it still reflects rather generic and sterile lab conditions, limiting its relevance to highly task-specific scenarios. Moreover, we did not directly assess participants’ sense of presence via standardized tools like the ITC-Sense of Presence Inventory (Lessiter et al., 2001) or the Slater-Usoh-Steed questionnaire (Usoh et al., 2000), which is an important component of ecological validity in VR. These aspects were outside the scope of our study, but their absence limits the study's ability to fully evaluate potential VR capabilities.

Several additional methodological limitations warrant consideration. To begin with, participants completed only a limited number of training trials per task, which may have been insufficient for full task familiarization and could have led to learning effects. Combined with the fixed task order, these factors may have influenced performance and contributed to the observed differences between tasks. Additionally, the use of a Bluetooth controller without precise latency benchmarking makes it difficult to determine whether the slower RTs observed in the VR-RT test reflect genuine behavioral differences or are due to technical delays. Finally, since the primary aim of this study was to examine the correlations between tests, we summarized participants’ single-trial results using measures of central tendency. This approach generated a large number of dependent variables (e.g., median RTs, ICV, etc.), increasing the potential risk of type I errors. More advanced statistical methods – such as multilevel modeling – could preserve trial-level resolution and account for variability across trials, tasks, and conditions, though these were beyond the scope of the present study.

### Future directions

Future research should continue exploring the applications of the VR-RT test across different populations and contexts. Longitudinal studies could examine the test’s sensitivity to changes in cognitive and motor functions over time, particularly in clinical populations. Additionally, future work could build on the current VR-RT test framework by developing more complex tasks and incorporating more specific and lifelike scenarios. Such studies might also address some of the current study’s limitations, for example, by including measures of presence, verifying system timing accuracy, extending familiarization periods, counterbalancing task order, or using advanced statistical approaches that retain single-trial resolution while accounting for within-subject variability across trials, tasks, and conditions.

It would also be beneficial to explore the advantages of collecting and analyzing additional performance indicators accessible via VR systems, such as movement trajectory, speed, acceleration, and eye-tracking data. Moreover, future studies could investigate the neural mechanisms underlying performance differences across VR tasks using neuroimaging techniques, providing deeper insight into the cognitive and motor processes at play.

## Conclusion

This study provides strong evidence supporting the validity of a novel VR-based RT assessment. The VR-RT test demonstrated strong correlations with traditional computerized RT measures while enabling the assessment of RT, MT, and MV in more dynamic and complex scenarios. Although the two tests yielded consistent relative performance, their results are not directly comparable, limiting the possibility of substituting one for the other. These findings highlight the potential of VR technology to revolutionize the assessment of cognitive and motor functions, paving the way for more lifelike and comprehensive testing methods in both research and practical settings.

## Supplementary Information

Below is the link to the electronic supplementary material.

Supplementary file1 (DOCX 253 KB)

## Data Availability

Materials and statistical analysis code are available in the OSF repository, at https://osf.io/2kg7n/?view_only=ca186012f42c4edaa445106fea0f27e2.

## Code availability

The COM-RT test, the novel VR-RT test, and the accompanying materials are available in the OSF repository, at https://osf.io/2kg7n/?view_only=ca186012f42c4edaa445106fea0f27e2.
