# Supplementary material for: A novel reaction time assessment in virtual reality: Advantages over computerized tests
Source: Behav Res Methods. 2025 Jul 16;57(8):227. doi: 10.3758/s13428-025-02752-w (PMC12267386; doi:10.3758/s13428-025-02752-w)
Supplement: Supplementary file 1 — Supplementary file1 (DOCX 253 KB) [file 13428_2025_2752_MOESM1_ESM.docx]

A Novel Reaction Time Assessment in Virtual Reality: Advantages Over Computerized Tests. Loushy Kay, Been & Pick.

## Supplementary Table 1: Distribution of movement time data of all participants

| Range | Movement time values  out of all sample values  Number (%) | Movement time o**utliers**  **out of all sample outliers**  Number (%) |
| --- | --- | --- |
| 12-49 | 4 (0.11%) | 0 (0%) |
| 50-99 | 335 (9.47%) | 0 (0%) |
| 100-199 | 2082 (58.83%) | 0 (0%) |
| 200-299 | 785 (22.18%) | 0 (0%) |
| 300-399 | 192 (5.43%) | 0 (0%) |
| 400-499 | 32 (0.9%) | 1 (1.25%) |
| 500-599 | 17 (0.48%) | 4 (5%) |
| 600-699 | 8 (0.23%) | 4 (5%) |
| 700-799 | 22 (0.62%) | 18 (22.5%) |
| 800-899 | 18 (0.51%) | 16 (20%) |
| 900-1507 | 44 (1.24%) | 37 (46.25%) |
| **Total** | **3539 (100%)** | **80 (100%)** |

Number (%) is presented for nominal variables; **Movement time** outliers were defined, for each participant, as values exceeding three times the average movement time of all other trials within a task; **The** **red line** represents the upper cut-off value of 600 ms, which excluded only 2.6% of the data (81.5% of these excluded values were classified as outliers); **A lower cut-off** was not established, as it lacked a clear functional rationale. Additionally, very few trials had extremely short movement times, and most of these were recorded from the same participants, who were athletes. This suggests that these shorter movement times likely reflect genuine values, and excluding them might eliminate valid data critical for studying more athletic populations.

## Supplementary Table 2: Correlations between virtual reality tasks for each variable: reaction time, movement time, and movement velocity

| **SRT^M^** | | | |
| --- | --- | --- | --- |
|  | Center | Spatial | Dynamic |
| Press | 0.486* | 0.285 | 0.231 |
| Center | - | 0.748* | 0.525* |
| Spatial | - | - | 0.794* |
| **SRT^A^** | | | |
|  | Center | Spatial | Dynamic |
| Press | 0.515* | 0.349 | 0.261 |
| Center | - | 0.703* | 0.531* |
| Spatial | - | - | 0.827* |
| **MT^M^** | | | |
|  | Center | Spatial | Dynamic |
| Center | - | 0.849* | 0.712* |
| Spatial | - | - | 0.713* |
| **MT^A^** | | | |
|  | Center | Spatial | Dynamic |
| Center | - | 0.827* | 0.722* |
| Spatial | - | - | 0.792* |
| **MV^M^** | | | |
|  | Center | Spatial | Dynamic |
| Center | - | 0.902* | 0.822* |
| Spatial | - | - | 0.896* |
| **MV^A^** | | | |
|  | Center | Spatial | Dynamic |
| Center | - | 0.899* | 0.816* |
| Spatial | - | - | 0.883* |

Pearson's correlations (r) within each variable across the virtual reality tasks. SRT^M^ median simple reaction time; SRT^A^ average simple reaction time; MT^M^ median movement time; MT^A^ average movement time; MV^M^ median movement velocity; MV^A^ average mfovement velocity. Sample size: n=48 for the *Press*, *Center,* and *Spatial* tasks; n=47 for the *Dynamic* task due to malfunction and missing data from one participant. * correlation is significant at p<0.001 (adjusted α=0.0041)
